# Shortened Spadin Analogs Display Better TREK-1 Inhibition, *In Vivo* Stability and Antidepressant Activity

**DOI:** 10.3389/fphar.2017.00643

**Published:** 2017-09-12

**Authors:** Alaeddine Djillani, Mariel Pietri, Sébastien Moreno, Catherine Heurteaux, Jean Mazella, Marc Borsotto

**Affiliations:** Centre National de la Recherche Scientifique, Institut de Pharmacologie Moléculaire et Cellulaire, UMR7275, Université Côte d'Azur Valbonne, France

**Keywords:** spadin-analogs, TREK-1 channel, PE 22-28, neurogenesis, synaptogenesis, antidepressant

## Abstract

Depression is a devastating mental disorder that affects 20% of the population worldwide. Despite their proven efficacy, antidepressants present a delayed onset of action and serious adverse effects. Seven years ago, we described spadin (PE 12-28) as a promising endogenous peptide with antidepressant activity. Spadin specifically blocks the TREK-1 channel. Previously, we showed *in vivo* that, spadin activity disappeared beyond 7 h after administration. In order to improve *in vivo* spadin stability and bioavailability, we screened spadin analogs and derivatives. From the study of spadin blood degradation products, we designed a 7 amino-acid peptide, PE 22-28. *In vitro* studies on hTREK-1/HEK cells by using patch-clamp technique, showed that PE 22-28 displayed a better specificity and affinity for TREK-1 channel compared to spadin, IC_50_ of 0.12 nM vs. 40–60 nM for spadin. In the same conditions, we also pointed out that different modifications of its N or C-terminal ends maintained or abolished TREK-1 channel activity without affecting PE 22-28 affinity. *In vivo*, the antidepressant properties of PE 22-28 and its derivatives were demonstrated in behavioral models of depression, such as the forced swimming test. Mice treated with spadin-analogs showed a significant reduction of the immobility time. Moreover, in the novelty suppressed feeding test after a 4-day sub-chronic treatment PE 22-28 reduced significantly the latency to eat the food pellet. PE 22-28 and its analogs were able to induce neurogenesis after only a 4-day treatment with a prominent effect of the G/A-PE 22-28. On mouse cortical neurons, PE 22-28 and its derivatives enhanced synaptogenesis measured by the increase of PSD-95 expression level. Finally, the action duration of PE 22-28 and its analogs was largely improved in comparison with that of spadin, up to 23 h instead of 7 h. Taken together, our results demonstrated that PE 22-28 and its derivatives represent other promising molecules that could be an alternative to spadin in the treatment of depression.

## Introduction

Depression is one of the most common mood disorders that represents a heavy economic burden in industrialized countries (Smith, 2014). Severe depression affects 2–5% of US citizens and up to 20% of the population suffer from mild depression (Nestler et al., 2002; Maletic et al., 2007; Krishnan and Nestler, 2008; Kessler et al., 2012). Depression is a complex syndrome with a variety of causes mostly genetic and environmental (Nestler et al., 2002). One of the main hypotheses proposed to explain the physiopathology of depression is the monoamine hypothesis where depletion of three monoamines serotonin (5-HT), norepinephrine (NA) or dopamine (DA) is thought to cause depression (Hillhouse and Porter, 2015). Later, several antidepressant (AD) drugs were developed in the aim to restore the physiological synaptic levels of the three neurotransmitters (Hillhouse and Porter, 2015). Several types of AD drugs were and are still used in the treatment of depression. Initially, depression was mainly treated by tricyclics family and at lesser extent by monoamine oxidase inhibitors (MAOIs) and serotonin-norepinephrine re-uptake inhibitors (SNRIs). However, these classes of ADs exhibit numerous and serious side effects (Hirschfeld, 2012). To reduce the frequency and occurrence of these side effects, these drugs were replaced by a generation of ADs more specific and with lesser adverse effects. This includes serotonin-selective re-uptake inhibitors (SSRIs) and norepinephrine selective re-uptake inhibitors (NSRIs) that are widely used nowadays and recommended as first-line treatment for depression (Nestler et al., 2002; Cleare et al., 2015). SSRIs and NSRIs are thought to increase monoamine synaptic concentrations by inhibiting the re-uptake of 5-HT or NA by blocking their specific transporters, SERT and NAT, respectively (Kohler et al., 2016). These AD drugs are more tolerated but their efficacy on depressive patients is not really improved. On the other hand, the actual ADs like fluoxetine are only efficient after 3–4 weeks of treatment, a latency period that still remains unexplained (Nestler et al., 2002). Thus, it is necessary to discover and characterize new targets for new AD drugs. Recently, different fast-onset AD drugs have been described like ketamine, scopolamine or GLYX-13 (Ramaker and Dulawa, 2017). Nevertheless, these molecules, particularly ketamine produces a number of adverse effects (Katalinic et al., 2013). Multimodal drugs, such as vortioxetine or vilazodone are a new class of ADs latterly approved by the US Food and Drug Administration for the treatment of major depressive disorders (Wang et al., 2015; Sowa-Kucma et al., 2017). Nevertheless, these drugs have not represented a clear improvement of antidepressant efficacy, but vortioxetine showed beneficial effects in depression-related cognitive impairment whereas vilazodone appeared to induce minor sexual dysfunctions (Deardorff and Grossberg, 2014; Li et al., 2015; Thase et al., 2016). Despite this therapeutic arsenal, more than 30% of depressive patients never remit even after several classical treatments (Rush et al., 2006). An alternate for drug therapy in resistant patients is the electroconvulsive therapy (ECT). ECT is efficient in 50% of pharmacotherapy-resistant patients (Heijnen et al., 2010) but ECT also induces some adverse effects mainly in the cognitive processes (Semkovska and McLoughlin, 2010). Thus, the discovery of new AD drugs is challenging. Ten years ago, we have identified the selective two-pore domain potassium channel TREK-1 (TWIK-related potassium channel-1) as a potential target for depression treatment (Heurteaux et al., 2006; Borsotto et al., 2015). TREK-1 channels are ubiquitous potassium channels that play pivotal role in stabilizing membrane potential and thus prevent cellular excitability (Honore, 2007). TREK-1 channels are very particular K_2P_ channels since they are involved in many physiological and physiopathological processes, such as pain, epilepsy, stroke, and depression (Lauritzen et al., 2000; Alloui et al., 2006; Heurteaux et al., 2006). TREK-1 became also an attractive target in cardiovascular research because it plays an important role in atrial fibrillation, pulmonary arterial hypertension and ventricular arrhythmia (Wiedmann et al., 2016; Decher et al., 2017). In the field of depression, we showed in five different models of depression that deletion of *kcnk2* gene, which encodes for TREK-1 channels results in a depression-resistant phenotype associated with an enhanced serotonergic neurotransmission and an increased neurogenesis in the hippocampus (Heurteaux et al., 2006). These observations led us to search for potent TREK-1 blockers. Then, we identified spadin which derives from a larger peptide called propeptide (PE) (Mazella et al., 2010). PE is a 44 amino-acid that results from the post-translational maturation in the Golgi apparatus of sortilin, also known as the neurotensin receptor-3 (Mazella, 2001). Spadin is a fast-acting AD which does not produce any side effects on functions that are controlled by the TREK-1 channel (Moha Ou Maati et al., 2012). It is able to counteract depression in only 4 days when classical ADs require 3–4 weeks to be efficient (Mazella et al., 2010). Moreover, spadin blocks TREK-1 with higher affinity, IC_50_~ 40–60 nM vs. IC_50_~ 6 μM for the most used SSRI fluoxetine (Mazella et al., 2010; Moha ou Maati et al., 2011). However, in mice, the AD activity of spadin measured by FST disappears beyond 7 h after an acute ip administration (Veyssiere et al., 2015). In order to improve the spadin stability *in vivo*, we decided to search for analogs or derivatives of spadin. In a previous study we have identified two analogs, analogs 3 and 8, that were synthesized by using the retro-inverso technology (Veyssiere et al., 2015). Although the gain in term of affinity and action duration was about 20, it is not sufficient to make these analogs competitive in regard of their synthesis cost in comparison to spadin. Then, we decided to search for shortened analogs. We first studied whether or not spadin is degraded in the blood circulation. We identified two shortened peptides. By comparing their ability to inhibit TREK-1 channel expressed in the hTREK-1/HEK cell line (Moha ou Maati et al., 2011), we identified the shortest efficient sequence that only contained 7 amino-acid called PE 22-28. This peptide was used as a core sequence for preparing analogs by chemical modifications of its N- or C-terminus ends and also by substitution of amino-acids. As PE 22-28, some modified peptide-analogs displayed a better potency in blocking TREK-1 channel and more importantly, they have retained their AD properties when injected in acute or sub-chronic treatments.

## Materials and methods

### *In vitro* analysis

#### Cell lines

The human TREK-1/HEK cell line (Moha ou Maati et al., 2011) and the native HEK293 cell line were maintained in Dulbecco's Modified Eagle's Medium (DMEM) supplemented with 10% (v/v) heat-inactivated fetal bovine serum, 1% (v/v) penicillin-streptomycin, 1% Glutamax. For the hTREK-1/HEK cells, 0.5 mg/ml G418 were added to the medium.

Cells were incubated in a humidified atmosphere containing 5% CO_2_. For electrophysiological measurements, cells were plated at a density of 20,000 cells per 35 mm dish.

In order to study the effects of shortened spadin analogs on hTREK-2, hTASK-1, hTRAAK and hTRESK, the native HEK293 cells were transfected with DNAs corresponding to the channels using JetPEI (Polyplus-transfection, France) following the provider's instructions.

#### Electrophysiological measurements

Cells from the hTREK-1/HEK cell line were seeded at a density of 20,000 cells/35 mm dish. Electrophysiological recordings were performed 24–48 h after plating using the whole-cell configuration of patch-clamp technique. TREK-1 currents (*I*_TREK−1_) were recorded using RK400 patch-clamp amplifier (Axon Instrument, USA). They were low-pass filtered at 3 kHz and digitized at 10 kHz using a 12-bit analog-to-digital converter digidata (1322 series, Axon Instrument, USA). *I*_TREK−1_amplitudes were expressed as current densities [current amplitude (pA)/membrane capacitance (pF)]. The results were expressed as mean ± SEM (standard error of the mean).

Pipettes were pulled from borosilicate glass capillaries using a dual-stage micropipette puller (PC-10, Narishige); they had a resistance of 1.5–3 MΩ. Cells were continuously perfused using an external bath solution containing in mM: 150 NaCl, 5 KCl, 3 MgCl_2_, 1 CaCl_2_, and 10 HEPES. The bath solution was initially adjusted to pH 7.4 with NaOH. The intra-pipette solution contained in mM: 155 KCl, 3 MgCl_2_, 5 EGTA, and 10 HEPES adjusted to pH 7.2 with KOH. In order to measure *I*_TREK−1_, a cocktail of potassium channel blockers was added to the bath solution. This cocktail contained: 3 mM 4-AP (4-aminopyridine), 10 mM TEA (tetraethylammonium), 10 μM Glibenclamide, 100 nM Apamin, and 50 nM Charybdotoxin. Data acquisition was carried out using a computer (Dell Pentium) with pClamp software (Axon Instrument, USA). Whole-cell currents were generated by running a pulse or ramp protocol every 5 s from −100 to +60 mV with a holding potential maintained at −80 mV. To evaluate the inhibitory effect on TREK-1 channels of shortened spadin analogs compared with spadin, cells were first activated by 10 μM arachidonic acid (AA). Dose-response curves were realized to compare spadin-analog effects with spadin using Origin 8.6 (Northampton, MA, USA).

Patch-clamp recording data were analyzed using Clampfit (Molecular Devices, USA). *I* = f(V) curves were obtained from −100 to +60 mV ramp. Data were presented as mean ± SEM.

### *In vivo* analysis

#### Animals

Naïve male C57Bl/6J mice from 7 to 9 weeks old were used in all experiments (Janvier laboratory, Saint Berthevin, France). Mice were housed (10 animals per cage) under a 12-h light/12-h dark cycle (light on at 8:00 am) in a ventilated room at a temperature of 22 ± 1°C. Animals had free access to water and food (A03; SAFE, Augy, France). All experiments were conducted according to policies on the care and use of laboratory animals of the Society for Neuroscience, and also with respect to national laws on animal use. The local Ethics Committee (CIEPAL, N° 736-02) approved the experiments.

#### Chemicals

Spadin, PE 22-28 and PE 22-28 analogs were purchased from GeneCust Europe, Luxembourg. G418, Arachidonic acid (AA), 4-AP (4-aminopyridine), TEA (tetraethylammonium), Apamin and Charybdotoxin were purchased from Sigma-Aldrich, France, Glibenclamide was purchased from ICN Biomedicals (USA).

#### *In vitro* half-life time of spadin in serum

The half-life time of spadin was measured in serum by incubating 10 nmoles of the peptide with 200 μl of mouse serum in the absence or in the presence of the metalloprotease inhibitor 1–10 phenanthroline (1 mM) for various times (2, 5, 15, 30, and 60 min) at room temperature. Incubations were stopped by direct acidification (5 μl of 2.5 M HCl), loaded in C-18 sepack cartridges, lyophilized, resuspended in 20% acetonitrile before loading onto HPLC for analysis of PE degradation/stability. Elutions of HPLC products were carried out by means of a 50-min linear gradient of acetonitrile from 20 to 70% at a flow rate of 1 ml/min. The amount of the intact PE for each incubation time was expressed as the percent of the initial amount of spadin.

### Behavioral tests

#### Porsolt forced swim test (FST)

Mice were individually placed for 6 min in a non-escapable cylinder (30 cm height and 15 cm diameter) half-filled with water at 22 ± 1°C. The immobility time was manually measured only during the last 4 min. A mouse was considered immobile when it remained immobile with only slight movements in order to keep its head above water (Porsolt et al., 1977).

#### Novelty suppressed feeding test (NSF)

Mice were deprived from food for 24 h before the test. A food pellet was placed on a white platform in the center of a highly illuminated area (45 × 45 × 20 cm). The floor was covered with wooden bedding. Mice were placed in the corner of the arena, during a period of 10 min, the latency to start eating the pellet was measured (Santarelli et al., 2003).

#### Learned helplessness test (LHT)

The learned helplessness test consists in a 4-day training session and a single day test.

During the training session mice were exposed to 360 inescapable 2 s foot shocks, with an inter-trial interval of 8 s. A non-shocked group was exposed to the apparatus for the same duration but no shock was delivered.

The test consisted in 30 trials separated by a 30 s interval. One trial was defined as a 5 s period before shock onset and was terminated when the mouse moved to the second compartment or at the end of the shock onset. The latency to escape for each mouse during every trial was recorded (Caldarone et al., 2000).

### BrdU labeling

Twenty hours after the injections of 5-Bromo-2′-deoxyuridine (BrdU) (12 mg per animals administered in three bolus of 100 μL of a solution of 40 mg/mL of BrdU diluted in 0.9% NaCl), mice were anesthetized with isoflurane and transcardially perfused first with NaCl 0.9% and, second with 4% paraformaldehyde. Brains were cut in 40 μm sections, by using a vibratome (Leica), throughout the entire hippocampus. Eight slices, from bregma 3.3 to bregma 5.3, were taken to process the BrdU immunohistochemistry as previously described (Heurteaux et al., 2006). For each BrdU labeling, slices were first incubated with a mouse monoclonal anti-BrdU antibody (1/7,000, Becton Dinckinson). For chromogenic immunodetection, sections were incubated during 2 h in specific biotin-conjugated secondary antibodies (1/400; Vector Laboratories) followed by a peroxidase-avidin complex solution, to amplify the reaction. The peroxidase activity of immune complex was visualized with DAB staining using the VectaStain ABC kit according to the manufacturer's protocol (Vector Laboratories).

### Synaptogenesis

Mouse cortical neurons were treated with 0.1 μM of the indicated spadin analog for different times and homogenized in the Laemmli buffer and analyzed onto 10% SDS PAGE gels. Separated proteins were then transferred from gels onto nitrocellulose membranes (VWR, Fontenay-sous-Bois, France) and blocked with either 5% skim milk or 5% BSA as indicated in PBS for 30 min at room temperature. Membranes were incubated with antibodies directed against PSD-95, overnight at 4°C. Tubulin or β-actin contents were determined after stripping using a 1/1,000 dilution anti-tubulin or anti-β-actin antibodies (Sigma-Aldrich, Saint-Quentin Fallavier). After four washes in 0.1% Tween/PBS, secondary anti-mouse or anti-rabbit horseradish peroxidase-conjugated antibodies (1/10000, Amersham Biosciences, Orsay, France) were incubated for 1 h at room temperature. Proteins were detected with the ECL plus detection reagents (Amersham Biosciences, Orsay, France) using a LAS-3000 imaging system (Fujifilm, Düsseldorf, Germany).

Relative intensities of the labeled bands were analyzed by densitometric scanning using ImageJ software (Wayne Rasband, Bethesda, USA). PSD-95 expression was normalized using total tubulin or β-actin labeling.

### Statistical analysis

Data are presented as mean ± SEM of at least 3 independent experiments. In GraphPad Prism (GraphPad software, La Jolla, USA), statistical comparisons were performed using Student's *t*-test or ANOVA one-way. A result is considered as statistically significant when *p* < 0.05.

## Results

### Spadin degradation in the serum

**Figure 1:**
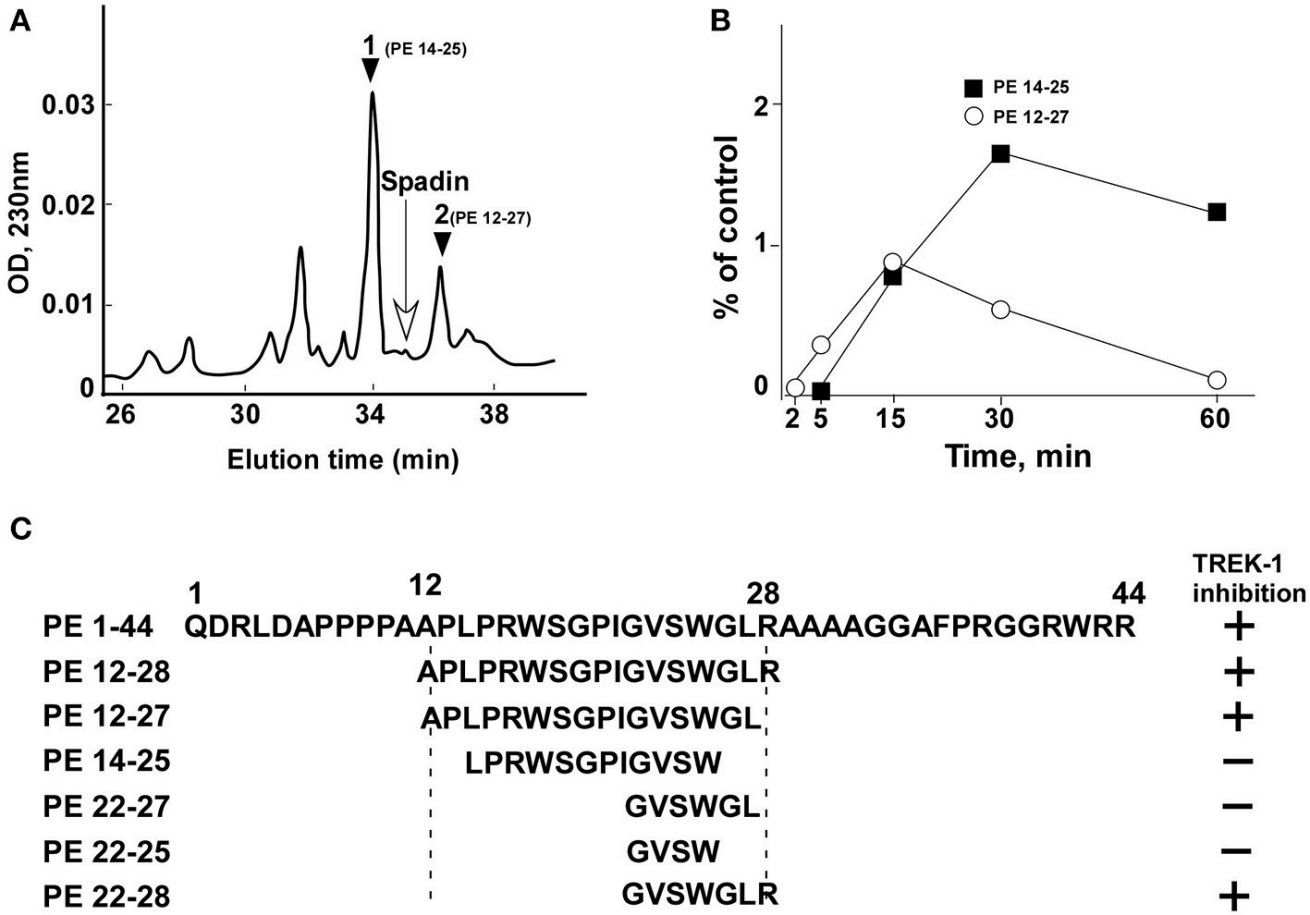
HPLC profiles of spadin degradation products in serum. **(A)** A typical HPLC profile obtained after 30 min incubation of spadin with mouse serum at 37°C. The position of spadin peak is indicated by an arrow. **(B)** Kinetics of appearance of peaks 1 (PE 14-28) and 2 (PE 12-27). **(C)** Sequences of shortened peptides and their capacity to inhibit TREK-1 activity compared with PE and spadin (PE 12-28) sequences.

From the analysis of spadin degradation after 30 min of incubation with serum at 37°C, we observed the disappearance of almost all spadin and the appearance of two other peaks, peak 1 and peak 2 (Figure 1A). Mass spectroscopy analyses indicated that peak 1 and peak 2 corresponded to sequences PE 14-25 and PE 12-27, respectively (Figure 1C). These peaks appeared rapidly and reached a maximal value at 15 min for peak 1 followed by a further degradation (Figure 1B). By contrast, peak 2 reached its maximal appearance at 30 min which was maintained up to 60 min (Figure 1B).

### Identification of the PE 22-28 sequence as the most efficient TREK-1 blocker with higher affinity compared to spadin

**Figure 2:**
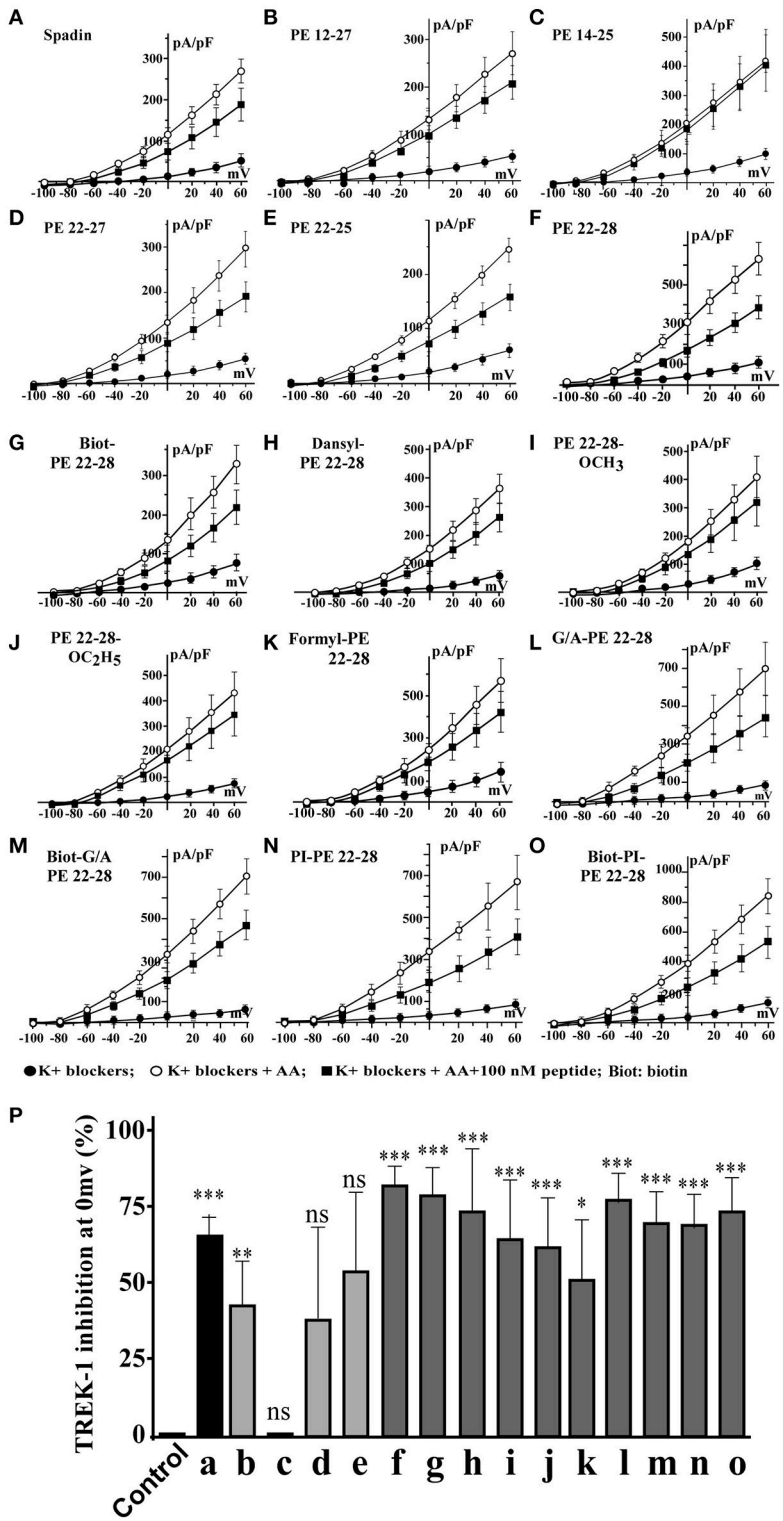
*I* = f(V) curves and % of TREK-1 inhibition. **(A–F)**, current density curves obtained with spadin and shortened peptides described in Figure 1C. **(G–O)**, current density curves obtained with PE 22-28 analogs. **(P)** Inhibition percentages of TREK-1 current measured at 0 mV for corresponding peptides described from “**A**” to “**O**”. Control value was obtained by using a solution of 0.9% NaCl. ns, not significant. ^*^p < 0.05, ^**^p < 0.01, ^***^p < 0.001.

Spadin shortened analogs were individually screened on the hTREK-1/HEK cell line (Moha ou Maati et al., 2011) using the patch-clamp technique (Figure 1C). Shortened PE peptides showed differences in their capacity to inhibit TREK-1 activity in comparison with spadin (PE 12-28) and PE 1-44 sequences. In all experiments (Figure 2), TREK-1 channels were prior activated with 10 μM AA (Patel et al., 1998). When the maximal amplitude was reached, we measured the ability of each peptide at 100 nM to inhibit the TREK-1 channel activity induced by AA and we compared them with spadin (Figure 2 and Table 1). We first tested the two peptides identified above (PE 14-25 and PE 12-27) (Figures 2B,C,P). No significant effect on TREK-1 channels was observed with the PE 14-25 analog (Figures 2C,P). The current density measured at 0 mV and compared with AA activity alone (100%) was 114.7 ± 10.6% (*n* = 8, *p* = 0.09). Interestingly, we found that PE 12-27 was able to strongly inhibit TREK-1 activity (28.4 ± 9.9%, *n* = 22, *p* = 0.03; Figures 2B,P).

**Table 1:** Spadin analogs and their ability to inhibit TREK-1 channel activity and reduce immobility times in FST.

**Figure 2 code**	**Peptide names**	**Modifications**	**% of TREK-1 inhibition**	***n***	***P***	**FST immobility times (s)**	***p***
a	PE 12-28 (Spadin)	Not modified	44.37 ± 8.817	7	0.0024	88.3 ± 7.0	0.0001
b	PE 12-27	Not modified	28.39 ± 9.916	22	0.0093	100.2 ± 5.0	0.0001
c	PE 14-25	Not modified	−14.73 ± 10.6	8	0.2074	112.2 ± 7.1	0.0080
d	PE 22-27	Not modified	25.7 ± 20.01	10	0.2311	168.2 ± 4.2	ns
e	PE 22-25	Not modified	36.02 ± 17.47	14	0.0599	100.2 ± 5.0	0.0001
f	**PE 22-28**	**Not modified**	**55.46** ± **4.555**	**13**	**0.0001**	**91.8** ± **6.1**	**0.0001**
g	Biotinylated-PE 22-28	Addition N-terminus	53.03 ± 6.416	12	0.0001	112.1 ± 4.3	0.0001
h	DansyI-PE 22-28	Addition N-terminus	48.78 ± 14.52	10	0.0084	104.6 ± 11.8	0.0010
i	PE 22-28-O-Methyl	Addition C-terminus	42.98 ± 13.47	12	0.0086	137.1 ± 8.1	0.0200
j	PE 22-28-O-Ethyl	Addition C-terminus	41.39 ± 11.52	10	0.0058	113.2 ± 8.5	0.0001
k	Formyl-PE 22-28	Addition N-terminus	32.45 ± 12.22	10	0.0262	ND	
l	**G/A-PE 22-28**	**Substitution-N- terminus**	**50.61** ± **7.935**	**10**	**0.0001**	**110.2** ± **3.6**	**0.0001**
m	**Biotinylated G/A-PE 22-28**	**Addition** + **substitution N-terminus**	**46.11** ± **7.743**	**11**	**0.0001**	**140.7** ± **7.1**	**0.0200**
n	PI-PE 22-28	Addition N-terminus	46.19 ± 7.565	7	0.0009	119.7 ± 11.8	0.0070
o	Biotinylated-PI-PE 22-28	Addition N-terminus	49.11 ± 7.454	10	0.0001	124.1 ± 11.7	0.0080
	Palmitoyl-PE 22-28	Addition N-terminus	26.69 ± 16.45	12	0.133	ND	
	FITC-PE 22-28	Addition N-terminus	22.1 ± 12.63	9	0.1183	ND	
	Acetyl-PE 22-28	Addition N-terminus	20.49 ± 8.777	15	0.035	ND	
	Myristoyl-PE 22-28	Addition N-terminus	18.04 ± 17.77	13	0.3302	ND	
	Long Chain biotinylated- PE 22-28	Addition N-terminus	15.86 ± 11.21	12	0.1847	ND	
	5'FAM-PE 22-28	Addition N-terminus	6.633 ± 7.065	11	0.3699	ND	
	F-Moc-PE 22-28	Addition N-terminus	5.826 ± 10.91	11	0.6051	ND	
	Stearic acid-PE 22-28	Addition N-terminus	5.412 ± 5.496	32	0.3399	ND	

*n, numbers of cells. In FST experiments, n, 10 mice for each peptide*.

*ns, not significant, ND, Not determined, p-values are from Student's t-test*.

*Bolded values correspond to the spadin-analogs retained for further studies*.

Then, we designed three other shortened peptides, PE 22-25, PE 22-27 and PE 22-28 (Table 1). Neither PE 22-27 (Figures 2D,P, Table 1) nor PE 22-25 (Figures 2E,P, Table 1) had significant effect in inhibiting *I*_TREK-1_ (27.5 ± 20%, *n* = 10, *p* = 0.23) and (36.02 ± 17.5%, *n* = 14, *p* = 0.06), respectively. Only, the PE 22-28 (Figures 2F,P, Table 1) was able to efficiently block TREK-1 activity (55.46 ± 4.6%, *n* = 13, *p* < 0.0001).

### Spadin-analog design

After the screening of these analogs with the hTREK-1/HEK cells, PE 22-28 was identified as the most efficient TREK-1 blocker and was retained for further studies. With the aim to increase again the stability and the efficacy of the peptide we used PE 22-28 as core peptide for the design of several analogs. Peptides that were able to block TREK-1 activity were described in the Figures 2G–P) and in Table 1. They include biotinylated PE 22-28, PI-PE 22-28, corresponding to the PE 20-28 sequence, biotinylated-PI-PE 22-28, G/A-PE 22-28 corresponding to the PE 22-28 sequence where the Glycine at position 22 was replaced by an Alanine residue, biotinylated-G/A-PE 22-28, dansyl-PE 22-28 where a dansyl chemical group was added at the N-terminus of the peptide, O-methyl-PE 22-28 and O-ethyl-PE 22-28 where a O-methyl or a O-ethyl chemical group was added to the C-terminus of the peptides, respectively.

We tested other analogs but they were unable to inhibit TREK-1 current (Table 1). Corresponding current-voltage curves are depicted in the Supplementary Figure 1.

**Figure 3:**
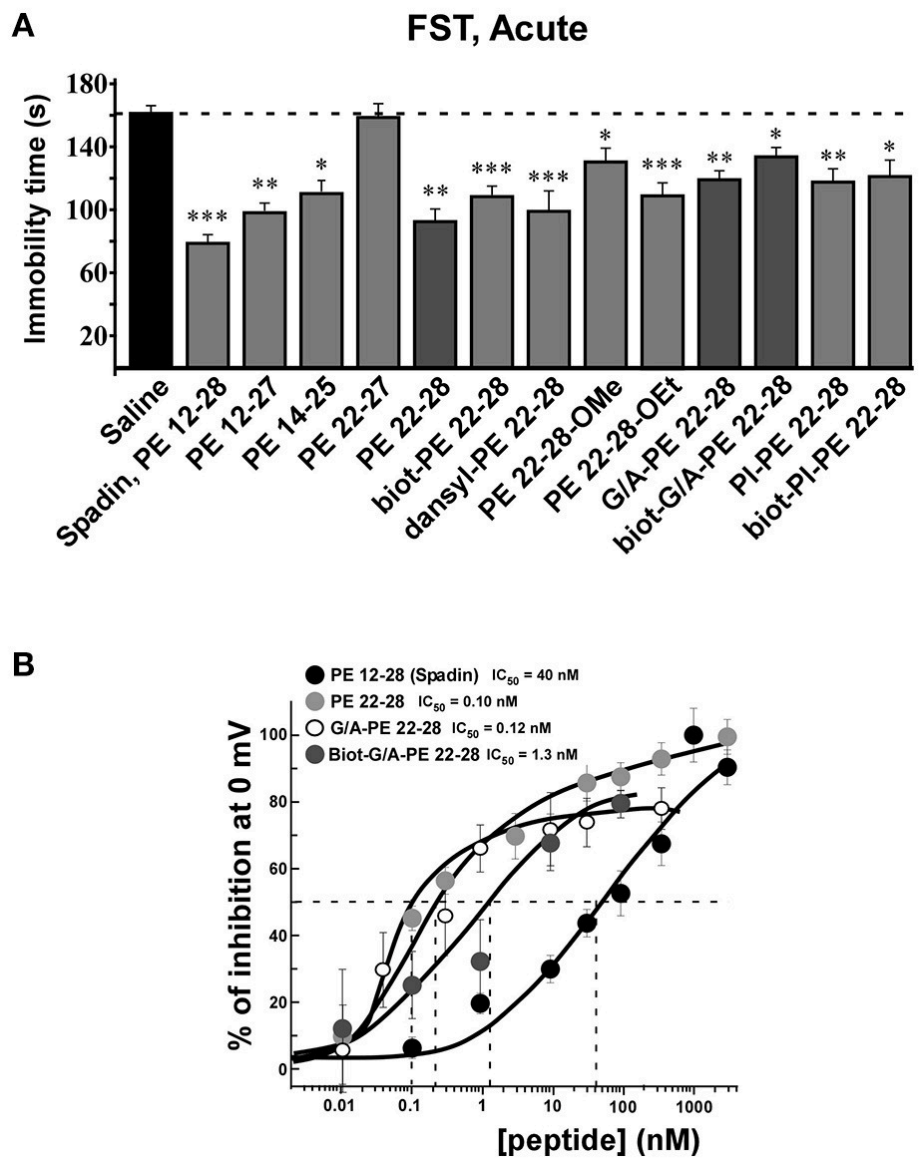
FST and dose-response curves of TREK-1 current inhibition by spadin-analogs. **(A)** Peptides able to inhibit TREK-1 channel activity were tested in the FST. Immobility times were measured 30 min after ip injection and compared with the immobility time obtained with saline injected mice. Spadin was injected at 100 μg/kg and spadin-analogs were injected at 3.2–4.0 μg/kg. ^*^p < 0.05, ^**^p < 0.01, ^***^p < 0.001. **(B)** Dose-response curves of TREK-1 current inhibition at 0 mV obtained with spadin-analogs compared with spadin. IC_50_ were 0.1, 0.12, 1.2, and 40.0 nM for PE 22-28, G/A-PE 22-28, biotinylated-G/A-PE 22-28 and spadin, respectively.

All screened analogs able to inhibit TREK-1 channel also displayed AD properties measured with the Forced Swim Test (FST) (Figure 3A, Table 1).

Analyses of electrophysiological and behavioral data allowed us to focus further studies on PE 22-28, G/A-PE 22-28 and biotinylated-G/A-PE 22-28. These three peptides are hereafter called spadin-analogs (bolded in Table 1). They shared common AD properties, a high percentage of TREK-1 current inhibition and, they had an affinity for TREK-1 that was largely increased in comparison to spadin (Figure 3B). The IC_50_ measured were 40, 0.12, 0.10, and 1.2 nM for PE 12-28 (Spadin), PE 22-28, G/A-PE 22-28 and biotinylated-G/A-PE 22-28, respectively.

### Spadin-analogs specifically block TREK-1 channel activity

**Figure 4:**
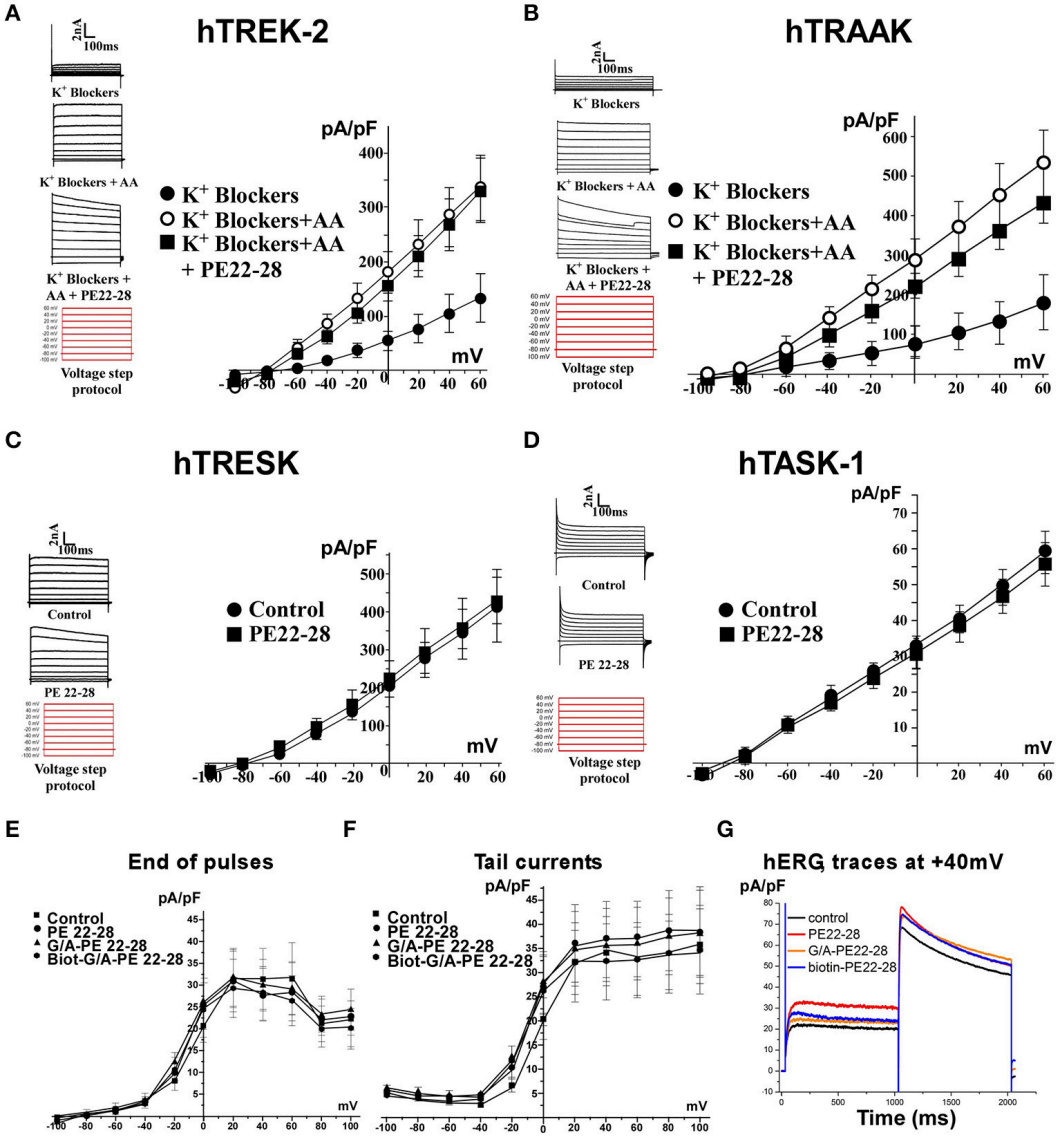
Spadin-analog specificity. **(A–D)** PE 22-28 was used as the representative peptide for testing the specificity of spadin-analogs vs. other K_2P_ channels, TREK-2 **(A)**, TRAAK **(B)**, TRESK **(C)**, and TASK-1 **(D)**. **(E–G)** Spadin-analogs were without effect on hERG channel activity. Current densities at the end of pulses **(E)**, current densities of tail currents **(F)**, and typical traces obtained at +40 mV with spadin-analogs **(G)**.

PE 22-28 was chosen as representative peptide among spadin-analogs and was tested on other K_2P_ channels like hTREK-2, hTRAAK, hTRESK, and also hTASK-1 (Lesage and Lazdunski, 2000; Kim et al., 2001; Talley et al., 2001; Lauritzen et al., 2003; Lafreniere et al., 2010). Native HEK cells were transfected by plasmids coding for these channels. TREK-2 and TRAAK were activated by 10 μM AA. Then 100 nM of PE 22-28 combined to AA were applied when current amplitude reached the maximum. PE 22-28 was inefficient in producing any change in the amplitude of the current (Figures 4A,B). Similarly, PE 22-28 was unable to modify currents generated by hTRESK and hTASK-1, two important K_2P_ channels in the brain (Figures 4C,D). Spadin-analogs represented by PE 22-28 did not inhibit these K_2P_ channels indicating that they are as specific as spadin for blocking TREK-1 channels.

More importantly, spadin-analogs tested at higher concentration (10 μM) did not modify the *I*_Kr_ current generated by hERG channels (Figures 4E–G). *I*_Kr_ current is the most important repolarizing current in the heart (Sanguinetti and Jurkiewicz, 1990; Cheng and Kodama, 2004). Dysfunction of these channels could cause death by Torsades de Pointes that are one of the most important side effects observed with AD drugs (Cheng and Kodama, 2004).

### Spadin-analogs display antidepressant properties in depression tests and in mouse model of depression

After an acute ip administration of 3.0–4.0 μg/kg spadin-analogs, the immobility time of mice was decreased significantly, 91.80 ± 6.1 s (*n* = 10, *p* < 0.0001), 110.2 ± 6.6 s (*n* = 10, *p* < 0.0001), and 140.7 ± 7.1 s (*n* = 10, *p* = 0.02) for PE 22-28, G/A-PE 22-28 and biotinylated-G/A-PE 22-28, respectively, values that have to be compared with that of the saline solution (161.7 ± 6.49 s) (Figure 3A).

**Figure 5:**
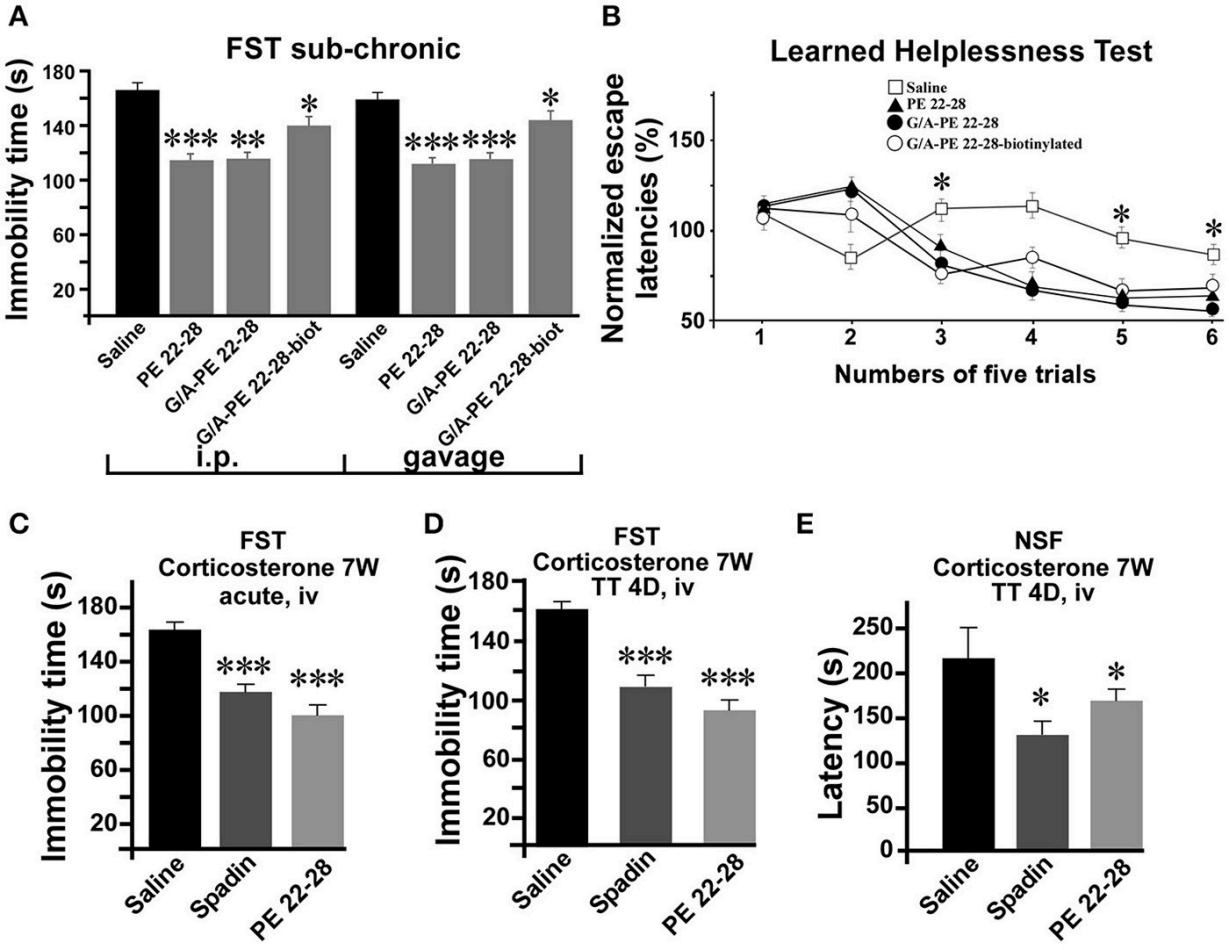
Behavior of spadin-analogs in mouse model of depression. **(A)** FST performed after a sub-chronic (4 days) treatment (3.0–4.0 μg/kg) with each spadin-analog or the 0.9% NaCl saline solution. Treatments were administered either by ip injection (100 μg/kg) or by gavage (1.0 mg/kg). **(B)** The Learned Helplessness test performed with each spadin-analog (3.0–4.0 μg/kg). 30 trials were divided in 6 pools of five trials. ^*^p < 0.05. **(C,D)** Corticosterone-induced model of depression. PE 22-28 was used as the representative peptide for spadin-analogs for comparing the effects of spadin-analogs with those of spadin in this chemically-induced mouse model. FST performed after acute (30 min after injection, **(C)** or sub-chronic (4 days treatment, **D**) ip injections of spadin (100 μg/kg) or PE 22-28 (3.0 μg/kg). Sub-chronic treatments administered at the same doses were also used before the NSF **(E)**. ^*^p < 0.05, ^**^p < 0.01, ^***^p < 0.001.

As spadin, spadin-analogs were efficient after sub-chronic treatments. Whether administered by ip injections (3.0 μg/kg) or gavage (1 mg/kg) (Figure 5A), they remained active in the FST. Then, we subjected mice to the Learned Helplessness Test, a validated and efficient test for identifying AD molecules. A 4-day sub-chronic treatment with spadin-analogs (3.0 μg/kg, ip) significantly reduced the escape latencies (Figure 5B). In the chemically induced model of depression using long term (7 weeks) corticosterone treatment (Zhao et al., 2008), PE 22-28 displayed the same AD properties as spadin after acute or sub-chronic treatments (3.0 μg/kg) (Figures 5C–E). In the FST, PE 22-28 (3.0 μg/kg, ip) was slightly more efficient in decreasing immobility time (98.1 ± 8.78 s, *n* = 10, *p* < 0.0001) than spadin (100 μg/kg; 117.4 ± 6.85 s, *n* = 10, *p* < 0.0001) in this model of depression in comparison with control (164.9 ± 6.03 s) (Figure 5C). Moreover, 4-day sub-chronic administrations of spadin-analogs were significantly efficient in decreasing the immobility time (89.60 ± 7.7 s, *n* = 10, *p* < 0.0001) compared to spadin (107.5 ± 6.5 s, *n* = 10, *p* < 0.0001) or saline (158.3 ± 7.15 s, *n* = 10; Figure 5D). These data confirmed the AD action of spadin-analogs on control or corticosterone induced depressive mice.

In the NSF, 4-day sub-chronic treatments with PE 22-28 (3 μg/kg) or spadin (100 μg/kg) significantly reduced the latency to eat the food pellet (129.2 ± 15.28 s, *n* = 10, *p* < 0.05) and (153.2 ± 5.41 s, *n* = 10, *p* < 0.05) in spadin and PE 22-28 groups, respectively in comparison with control (226.1 ± 34.97 s, *n* = 10) in the corticosterone induced model of depression (Figure 5E). The NSF test predicts not only depression but also neurogenesis (Duman et al., 2001; Santarelli et al., 2003). We hypothesized that spadin-analogs could increase neurogenesis in the cortex and the hippocampus.

### Spadin-analogs increase neurogenesis *in vivo* in the hippocampus after 4-day treatment

**Figure 6:**
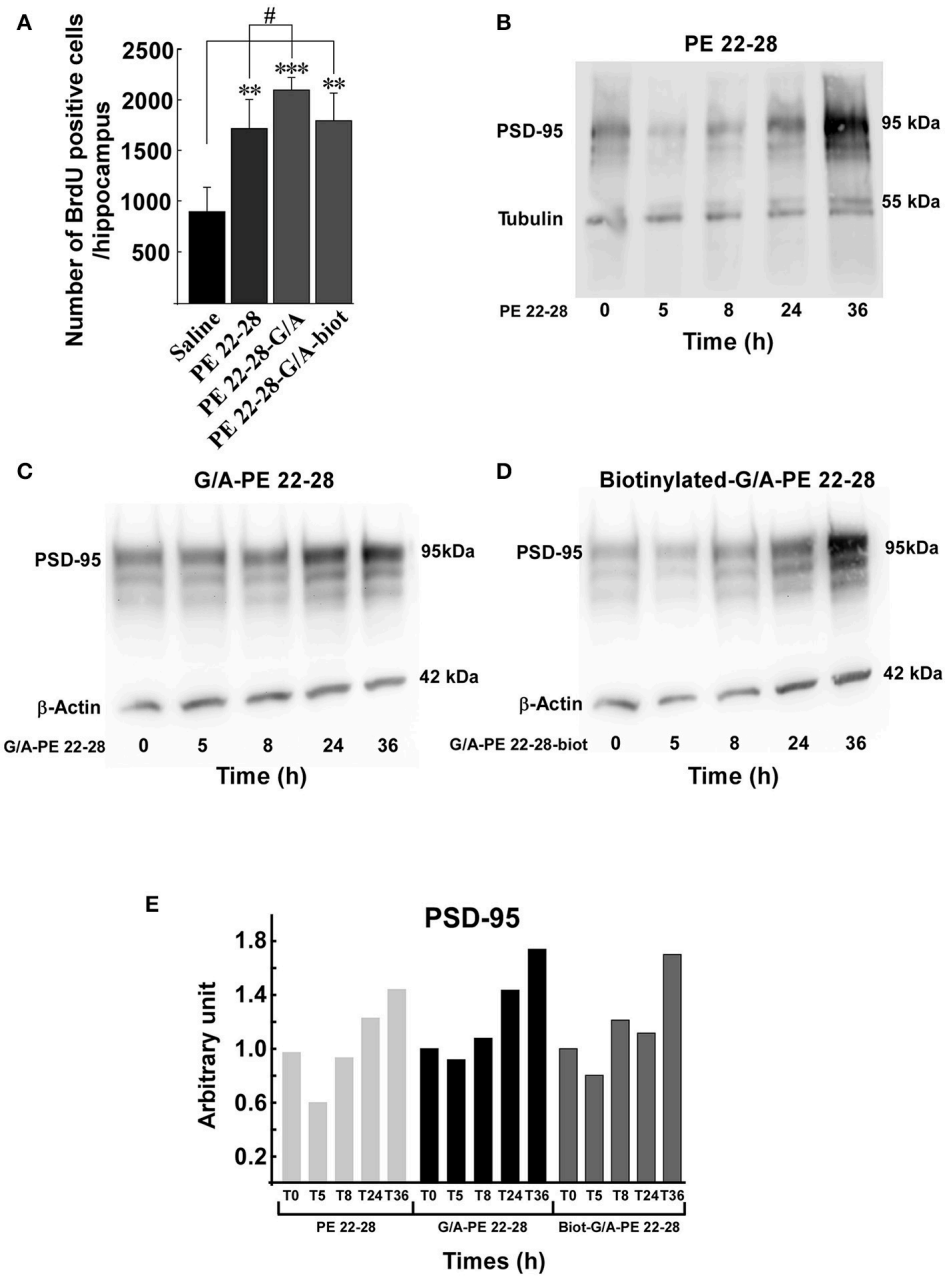
Neurogenesis and synaptogenesis. **(A)** Neurogenesis was assessed by measuring the number of BrdU positive cells per hippocampus after sub-chronic treatments (3.0–4.0 μg/kg, 4 days) with spadin-analogs. The cell number was given for the entirety of hippocampus. ^**^p < 0.01, ^***^p < 0.001, ^#^*p* < 0.05. **(B–D)** Synaptogenesis was assessed by measuring the increase in the level of PSD-95 in mouse cortical neuron. Mouse cortical neurons were treated with 0.1 μM of the indicated spadin-analog PE 22-28 **(B)**, G/A-PE 22-28 **(C)** and biotinylated-G/A-PE 22-28 **(D)**. At the indicated times neurons were homogenized in Laemmli buffer and submitted to Western blot analysis. **(E)** Histogram illustration of the PSD-95. For each spadin-analog, the PSD-95 amount at 36 h was about twice than that measured at 5 h.

Several studies demonstrated that a chronic AD treatment up-regulates neurogenesis in the hippocampus (Duman et al., 2001; Santarelli et al., 2003). We have previously shown that spadin increased neurogenesis and CREB activation in the hippocampus only after a 4-day treatment (Mazella et al., 2010). We wondered whether spadin-analogs produced the same effects. Mice were ip treated (3.0–4.0 μg/kg/day) for 4 days with spadin-analogs and, on the 5th day, were sacrificed. the 4-day treatment with spadin-analogs significantly increased BrdU positive cells (1736 ± 126 (*n* = 5, *p* < 0.0001), 2110 ± 132, (*n* = 5, *p* < 0.0001), 1809 ± 267 (*n* = 5, *p* < 0.0001), for PE 22-28, G/A-PE 22-28, and biotinylated-G/A-PE 22-28, respectively) in comparison with saline injected mice (899 ± 109, *n* = 5) (Figure 6A). These data confirmed that similarly to spadin, spadin-analogs have kept their ability to induce *in vivo* hippocampal neurogenesis.

### Spadin-analogs increase synaptogenesis *in vitro* in cortical neurons

Incubation of cortical neurons with 0.1 μM of spadin-analogs enhanced synaptogenesis as illustrated by the increase in the expression of PSD-95 36 h after incubation (Figures 6B–D). Except a slight decrease during the first 5 h, spadin-analog treatments continuously increased the expression of PSD-95 up to 36 h (Figure 6E). These data showed that spadin-analogs not only increased neurogenesis but also synaptogenesis, indicating that the fate of a majority of newborn cells is to generate mature neurons.

### Action duration of spadin-analogs

**Figure 7:**
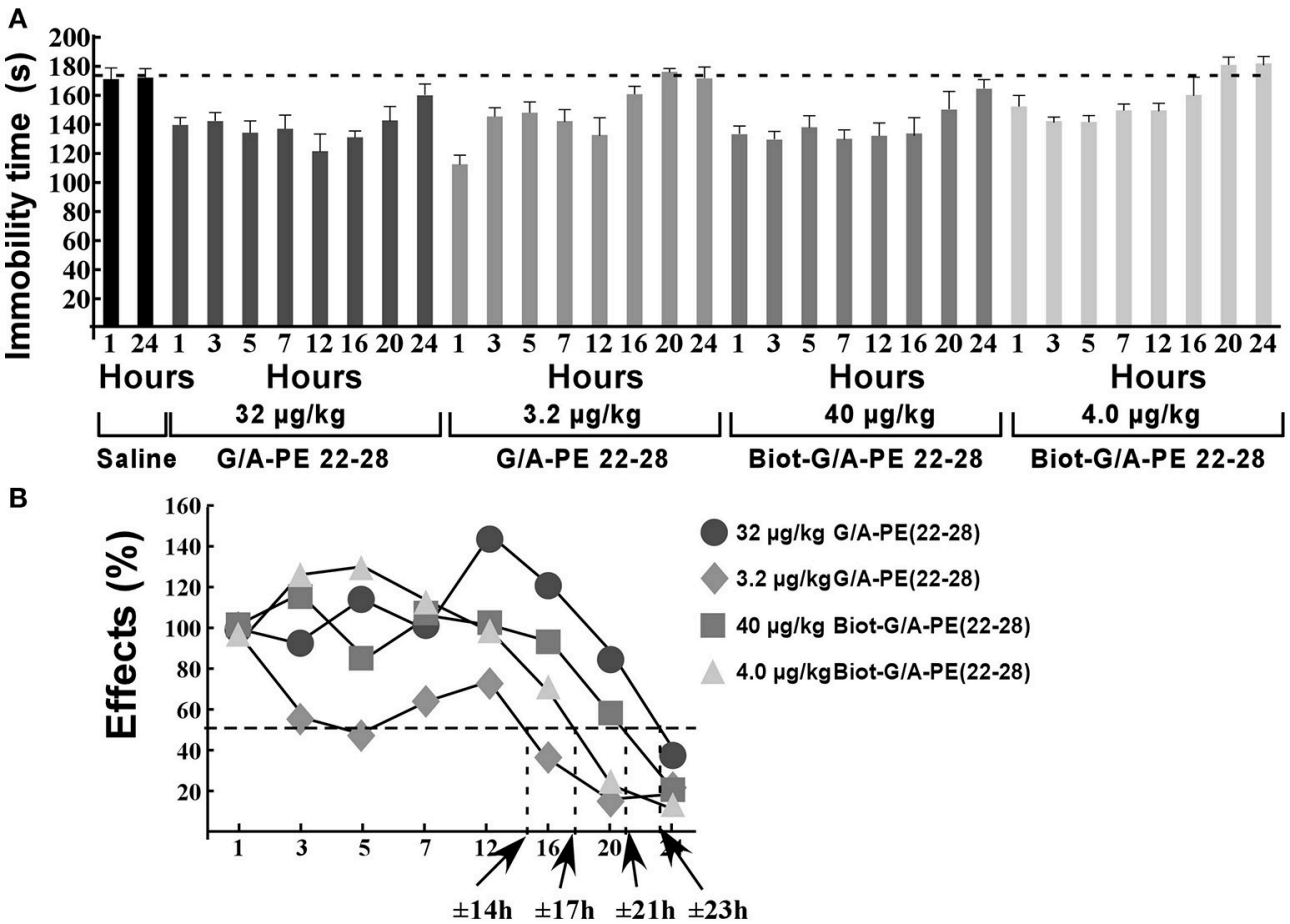
Action duration of G/A-PE 22-28 and biotinylated-G/A-PE 22-28 measured by FST. **(A)** At each time-point, 10 naïve mice were tested for immobility in the FST. Saline injected mice were tested only at two times, 1 and 24 h. For each spadin-analog, at time 1 h the difference between saline and spadin-analog treated mice was considered as 100%, Time-point immobility times were normalized to this 100% value. **(B)** Half-time effects were approximately of 14, 21, 17, and 23 h for 3.2 μg/kg of G/A-PE 22-28, 32 μg/kg of G/A-PE 22-28, 4.0 μg/kg of biotinylated-G/A-PE 22-28 and 40 μg/kg of biotinylated-G/A-PE 22-28, respectively.

Naïve mice (10 per time groups) were injected once to obtain a dose of 3.2 μg/kg or 32 μg/kg of G/A-PE 22-28 or a dose of 4.0 μg/kg or 40 μg/kg of biotinylated-G/A-PE 22-28. These doses were injected in a bolus of 100 μL of 0.9% NaCl. At times 1, 3, 5, 7, 12, 16, 20, and 24 h after injection, mice were submitted to FST (Figure 7A). Immobility times were compared to those obtained with mice injected with the saline solution (0.9% NaCl). FST for saline injected mice were only performed at times 1 and 24 h, immobility times were very similar, 171.2 ± 8.2 s and 175.5 ± 6.8 s, respectively (Figure 7A). To calculate the half-effect time, the immobility time measured at 1 h of saline injected mice was subtracted from the immobility time measured at 1 h for spadin-analogs, the difference value was considered as 100%. Other immobility times were normalized to the 100% value (Figure 7B). Calculated half-effect time values were 14, 17, 21, and 23 h for G/A-PE 22-28 (3.2 μg/kg), biotinylated-G/A-PE 22-28 (4.0 μg/kg), G/A-PE 22-28 (32 μg/kg) and biotinylated-G/A-PE 22-28 (40 μg/kg), respectively (Figure 7B). These values were higher than the one previously obtained with spadin (6 h) (Veyssiere et al., 2015).

## Discussion

### Spadin-analogs are more potent TREK-1 blockers than spadin

Depression is the most devastating and common mood disorder (Wong and Licinio, 2001). Treatments available nowadays target several proteins and undergo different mechanisms of action (Schechter et al., 2005). However, classical ADs are not fully specific and generally lead to side effects with different degree of severity. In order to avoid these adverse effects and improve the selectivity of the AD drugs, our strategy has consisted in focusing on improvement of the affinity, bioavailability and efficacy of the endogenous peptide that we have previously identified and called spadin (Mazella et al., 2010). Spadin was designed from a larger peptide called PE, a 44 aa peptide released in the blood flow following the translational maturation of the sortilin or neurotensin receptor 3 (Munck Petersen et al., 1999; Mazella, 2001). First, we identified degradation products of PE in the blood. From the peptides we identified we have designed a short 7 aa peptide, called PE-22-28. It displayed a better affinity for the TREK-1 potassium channel, a target that we have previously identified in the depression mechanism (Heurteaux et al., 2006). PE 22-28 was used for the design of 16 analogs. Because increasing the drug crossing through the blood brain barrier is a crucial goal for a therapeutic drug acting on brain targets and, because it was shown that biotinylation can increase peptide brain uptake, we synthesized some biotinylated derivatives (Scherrmann, 2002; Wu et al., 2002). By testing the different PE 22-28 derived peptides for their ability to inhibit TREK-1 channels expressed in the hTREK-1/HEK cell line (Moha ou Maati et al., 2011), we only retained those that inhibited more than 35% of TREK-1 activity in order to measure their AD properties by FST. Then, by comparing both abilities, we only retained 3 peptides (that we called spadin-analogs) PE 22-28, G/A-PE 22-28 and biotinylated-G/A-PE 22-28 for further studies.

Here, we showed that spadin-analogs displayed higher potencies in blocking TREK-1 channels when compared with spadin. Their IC_50_ were increased by more than 300 fold, 0.10 nM, and 0.12 nM for PE 22-28 and G/A-PE 22-28, respectively, these values have to be compared with spadin affinity, 40–60 nM (Mazella et al., 2010; Moha Ou Maati et al., 2012).

### Spadin-analogs are specific for the TREK-1 channel

Additionally, spadin-analogs have kept their specificity for TREK-1 channels (Moha Ou Maati et al., 2012). They were unable to inhibit TREK-2 and TRAAK channels, the two other members of the TREK channel sub-family (Kim et al., 2001; Honore, 2007). The specificity of spadin and its analogs for TREK-1 channel could be accounted by the sequence differences between the three channels: TREK-1 and TREK-2 share 63% of identity and only 45% between TREK-1 and TRAAK (Noel et al., 2011). They were also unable to inhibit TRESK (Lafreniere et al., 2010; Wood, 2010) and TASK-1 (Lauritzen et al., 2003) channels, two K_2P_ channels that are important in the brain and, as TREK-1 they are both modulated by volatile anaesthetics (Patel and Honore, 2001). Here again, sequence differences could account for the absence of effects, homologies between TREK-1 and both channels are around 50% (Noel et al., 2011). More noteworthy, spadin-analogs were devoid of effects on hERG channels that are responsible for the cardiac *I*_Kr_ current, one of the main potassium repolarizing current in the cardiac ventricle (Sanguinetti and Jurkiewicz, 1990; Cheng and Kodama, 2004).

Spadin and its analogs block TREK-1 channels more efficiently when they are activated by arachidonic acid indicating the need of an open-state conformation of the channel. The weak direct inhibition of TREK-1 in basal condition could be due to the necessity for spadin to access the selectivity filter in a closed channel.

The monoamine hypothesis of depression was expanded to other recent hypotheses mainly the neurotrophic and neurogenesis hypothesis that suggest that a decrease in neurotrophic factors, such as the brain-derived neurotrophic factor (BDNF) or in adult hippocampal neurogenesis are in one way or another associated with depression (Yohn et al., 2017). Classical ADs take several weeks to produce antidepressant activity, a mechanism that is thought to be mediated through neurogenesis (Santarelli et al., 2003; Malberg and Schechter, 2005). Interestingly, spadin and analogs display their antidepressant response within 4-day treatment, this short time correlates with the same period required for hippocampal neurogenesis to develop (Devader et al., 2015). The rapid increase in the BDNF expression in the hippocampus after *in vivo* administration of spadin points out the fact that spadin and derivative peptides induce a fast expression of BDNF to be distinguished from the slow BDNF expression observed with the conventional ADs. Two phases, fast and slow, are also observed with ketamine (Kavalali and Monteggia, 2015). Nevertheless, cellular pathway of neurogenesis activation are different. Ketamine uses the mTOR pathway (Kavalali and Monteggia, 2015) whereas spadin does not interfere with mTOR signaling (Devader et al., 2015).

### Spadin-analogs are potent antidepressants

Spadin-analogs have also kept the AD properties of spadin. We showed that spadin-analogs behaved as an AD drug in the FST but also in the learned helplessness test. Both tests are commonly and widely used by pharmaceutical industries for characterizing new AD drugs. Importantly, we showed that spadin-analogs were also efficient after gavage, indicating that these molecules could be administered *per os*. Interestingly, in a chemically (corticosterone)-induced model of depression spadin-analogs were as efficient as spadin for decreasing depression-like behavior generated by the corticosterone treatment. As expected for spadin derivatives, spadin-analogs were efficient after only 4 days of treatment. This unique property is crucial because it considerably reduces the onset time to observe the efficiency of the AD treatment. That is particularly interesting because the majority of suicides occurs during the first weeks following an AD treatment (Moller, 2003).

### Spadin-analogs are more stable than spadin *in vivo*

Another remarkable property of spadin-analogs is their prolonged action duration. Despite a relatively short *in vitro* serum half-life time, the *in vivo* antidepressant efficacy measured by FST lasted for almost 24 h. G/A-PE 22-28 or biotinylated-G/A-PE 22-28 injected at doses as low as 32.0 or 40.0 μg/kg, respectively had a half-time of effect of 23 and 21 h after injection. These values represent a huge improvement in comparison with spadin. In the same conditions, the half-time effect of spadin at a dose of 100 μg/kg was only of 6 h (Veyssiere et al., 2015).

Chronic treatments with ADs are known to induce neurogenesis in the hippocampus (Duman et al., 2001; Malberg and Schechter, 2005). However, neurogenesis up-regulation currently occurs after 2–4 weeks of administration. This is consistent with the fact that classical ADs are efficient only after the same period. Previously, we have shown that spadin was able to increase BrdU incorporation and CREB activation after only 4 days of treatment (Mazella et al., 2010). *In vivo* administration of spadin induces neurogenesis over different time scale through two phases, a rapid increase in the expression of BDNF and a slow spine maturation (Devader et al., 2015). Since targets and specificity for these targets are the same between spadin and its derivatives, we could speculate that spadin analogs could behave identically to spadin. Here, we demonstrated that spadin-analogs were also able to induce neurogenesis in the dentate gyrus. Interestingly, spadin-analog treatments also increased the PSD-95 expression, a biomarker of synaptogenesis. These observations indicated that number of newborn neurons were functional and should participate to the brain network. This property is crucial for the AD action of spadin-analogs.

## Conclusion

Our final goal is to make spadin-analogs drugs usable in clinics. All our data converge toward this goal. Spadin-analogs are specific for the TREK-1 channel, a target in the depression pathway, and efficient as AD. They induce neurogenesis after only 4 days of treatment. We have demonstrated that spadin and its retro-inverso analogs have no deleterious effects on pain, ischemia or at the cardiac levels (Moha Ou Maati et al., 2012). Since spadin analogs share the same targets (TREK-1 and NTR-3) and, have no effect on the other K_2P_ channels, more importantly they do not modify hERG channel activity then, we could expect that spadin analogs would be devoid of side effects. The absence of adverse effects differentiates spadin-analogs from other antidepressant drugs, such as SSRIs, SNRIs, tricyclics (Ferguson, 2001), or ketamine (Katalinic et al., 2013). Additionally to the shared properties with spadin, spadin-analogs display a largely increased affinity for TREK-1 and, also a largely increased action duration. Another important point concerns the shortening of the peptide that will induce a lower cost for the drug manufacturing and *in fine* a decrease of economic burden to treat depressive patients. All these reasons encourage us to think that in the really near future spadin-analogs will constitute a promising alternative to spadin and become efficient AD drugs usable in clinic.

## Author contributions

AD, performed electrophysiological experiments. MP, performed behavioral experiments. SM, performed biochemical experiments. CH, JM, and MB, conceived and designed the experiments. CH, JM, and MB, contributed reagents/materials/analysis tools and wrote the paper.

### Conflict of interest statement

The authors declare that the research was conducted in the absence of any commercial or financial relationships that could be construed as a potential conflict of interest.

## Abbreviations

AD: antidepressant
FST: Forced Swim Test
NSF: Novelty-suppressed Feeding
LHT: Learned Helplessness Test
ip: intraperitoneal
PSD-95: Post-Synaptic Density protein 95.
