# Supplementary material for: Shortened Spadin Analogs Display Better TREK-1 Inhibition, In Vivo Stability and Antidepressant Activity
Source: Front Pharmacol. 2017 Sep 12;8:643. doi: 10.3389/fphar.2017.00643 (PMC5601071; doi:10.3389/fphar.2017.00643)
Supplement: Supplementary file 1 [file DataSheet1.DOC]

**Shortened spadin analogs display better TREK-1 inhibition, *in vivo* stability and antidepressant activity**

Alaeddine DJILLANI^1,*^, Mariel PIETRI^1,*^, Sébastien MORENO^1^, Catherine HEURTEAUX^1^, Jean MAZELLA^1^, Marc BORSOTTO^1#^

**Supplementary Figure 1**


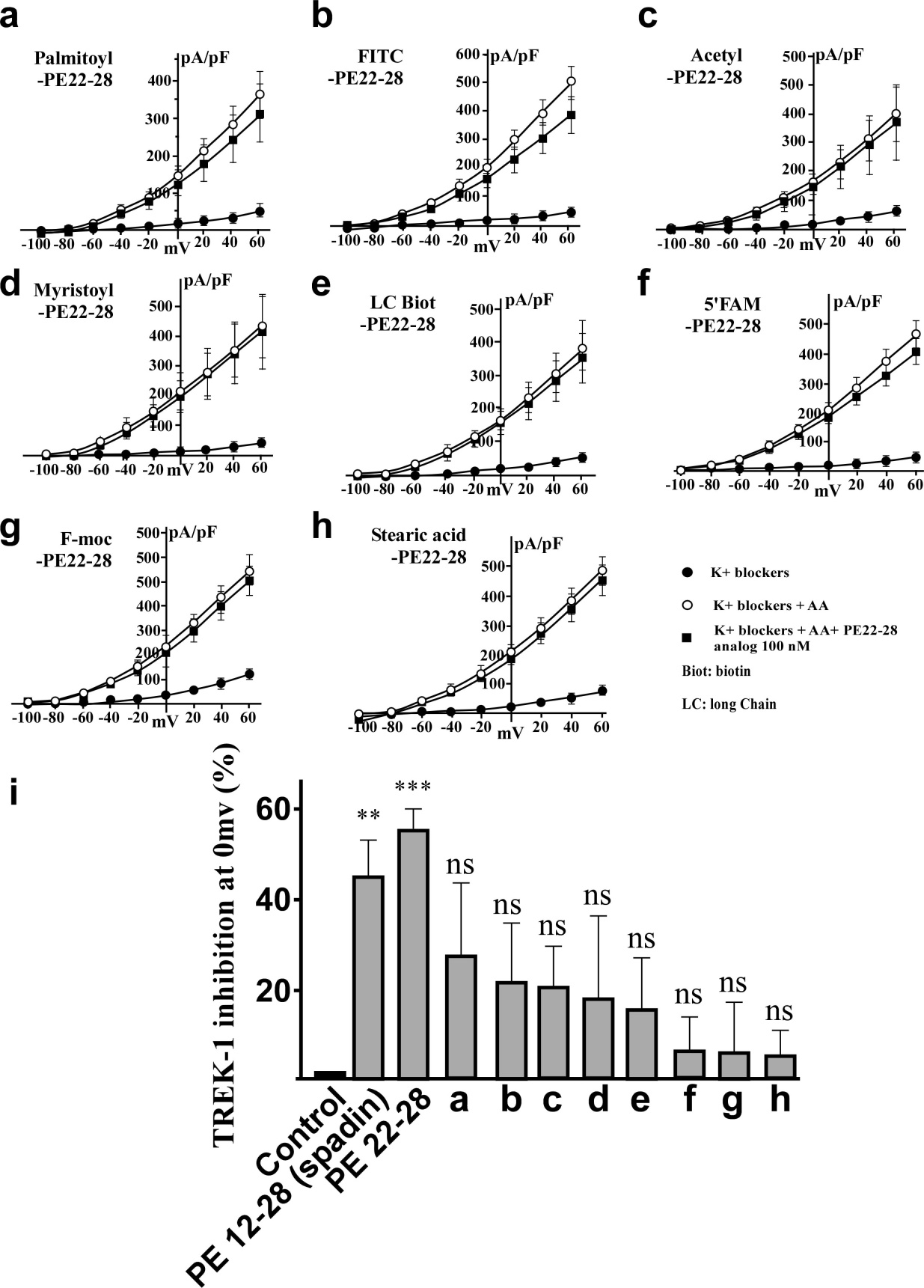


Inactive PE 22-28 analogs. **a-h**- Current densities = f(mV) curves for each analog. **i**- Percentage of TREK-1 inhibition at 0mV. ns, not significant.
